# Expansion of Gammadelta T Cells from Cord Blood: A Therapeutical Possibility

**DOI:** 10.1155/2018/8529104

**Published:** 2018-03-07

**Authors:** Sofia Berglund, Ahmed Gaballa, Piamsiri Sawaisorn, Berit Sundberg, Michael Uhlin

**Affiliations:** ^1^Department of Medicine, Solna, Karolinska Institutet, Stockholm, Sweden; ^2^Department of Clinical Science, Intervention and Technology, Karolinska Institutet, Stockholm, Sweden; ^3^Center for Research and Innovation, Faculty of Medical Technology, Mahidol University, Bangkok, Thailand; ^4^Department of Applied Physics, Royal Institute of Technology, Stockholm, Sweden; ^5^Department of Immunology and Transfusion Medicine, Karolinska University Hospital, Stockholm, Sweden

## Abstract

Gammadelta (*γδ*) T cells are found in both blood and tissues and have antiviral and antitumor properties. The frequency of *γδ* T cells in umbilical cord blood (UCB) is low, and the majority express *δ*1, in contrast to blood, whereas the main subset is δ2*γ*9 T cells. UCB γδ T cells are functionally immature, which together with their scarcity complicates the development of UCB *γδ* T cell therapies. We aimed to develop an effective expansion protocol for UCB γ*δ* T cells based on zoledronate and IL-2. We found that culture with 5 *μ*M zoledronate and 200 IU IL-2/ml medium for 14 days promoted extensive proliferation. The majority of the cultured cells were *γ*9*δ*2 T cells. The fold expansion of this, originally infrequent, subset was impressive (median and maximum fold change 253 and 1085, resp.). After culture, the cells had a polyclonal *γδ* T cell repertoire and the main memory subset was central memory (CD45RO^+^ CD27^+^). The cells produced cytokines such as IL-1B, IL-2, and IL-8 and displayed significant tumor-killing capacity. These results show that development of *in vitro* expanded UCB *γδ* T cell therapies is feasible. It could prove a valuable treatment modality for patients after umbilical cord blood transplantation.

## 1. Introduction

*γδ* T cells constitute a unique minor subpopulation of T cells. Their features place them between innate and adaptive immunity [1] and include antigen recognition independent of major histocompatibility complex (MHC) presentation, cytokine production, and cytotoxicity [2–4].

In humans, there are several subsets of *γδ* T cells, identified by the combination of specific TCR *γ* and *δ* chains. The major *γδ* T cell population in peripheral blood (PB) expresses a TCR containing *δ*2 and *γ*9 chains [4]. This subset, termed *δ*2*γ*9 T cells, recognizes phosphoantigens, phosphorylated nonpeptidic metabolic intermediates of the isoprenoid biosynthesis [5] in both invading microbes and the body's own cells, in an MHC-unrestricted manner [4, 6]. Endogenous phosphoantigens are upregulated in cellular dysregulation, a state that can result from infection or malignant transformation, and have been implicated as a key factor in *γδ* T cell tumor recognition [4, 7, 8]. This indicates a role for *δ*2*γ*9 T cells in anticancer immunity.

Another important *γδ* T cell subset expresses the *δ*1 chain and is known to be predominant in the thymus and peripheral tissues. These *γδ* T cells are considered to recognize various stress-related antigens, most of which are uncharacterized. Known specificities include CD1 family proteins [9], MICA, and MICB [10, 11].

*γδ* T cells constitute approximately 5% of circulating T cells in adult PB [12], but the compartment can increase substantially in certain situations [13, 14]. In umbilical cord blood (UCB), *γδ* T cells are present at a low frequency (<1% of lymphocytes [15]) and express a naïve phenotype. The repertoire is polyclonal, with *δ*1 T cells being the predominant subtype [16, 17]. The *δ*2*γ*9 T cells are few in UCB [18, 19] and have been described as functionally immature: they express low frequencies of high-affinity interleukin 2 receptor (IL-2R) *β* chain and have reduced interferon- (IFN-) *γ* production [20]. However, a higher expression of the IL-2R *α* chain has been reported in UCB *δ*2*γ*9 T cells [20], as well as a decreased expression of the common IL-2R*γ* chain on UCB lymphocytes in general [21].

*γδ* T cell immunotherapy is currently being explored. An important milestone was the discovery that bisphosphonates, drugs for osteoporosis, inhibit a downstream enzyme in the isoprenoid biosynthesis, causing accumulation of metabolites and making exposed cells *γ*9*δ*2 T cell targets. Thus, *in vitro* expansion of *γ*9*δ*2 T cells can be easily performed with clinically approved and readily available compounds.

Expansion of *γδ* T cells from adult PB has been explored with considerable success [22–24], and several early clinical trials of expanded PB *δ*2*γ*9 T cell immunotherapy have been performed in patients with malignancies [25]. Other trials have explored treatment of patients with bisphosphonates and IL-2 to induce *in vivo γ*9*δ*2 T cell expansion [25]. Noteworthy results include those of a phase I/II study of *in vitro* expanded *γ*9*δ*2 T cell therapy in patients with renal cell carcinoma [26], where reduction of lesions or significantly reduced tumor growth rate could be seen in 6/11 patients. A commercial product with *in vitro* expanded V*δ*2V*γ*9 T cell has been developed and found safe in a phase I study in patients with renal cell carcinoma [27, 28].

*In vitro* expansion of *γδ* T cells from umbilical cord blood (UCB) for clinical use includes several challenges, including the low number of *γδ* T cells present, the low percentage of *γ*9*δ*2 T cells capable of responding to phosphoantigens, and their immature phenotype. UCB *γδ* T cells have been found to be relatively unresponsive to model phosphoantigens, but to proliferate in response to bisphosphonates [15, 18].

IL-2 and IL-15 have been used in combination with bisphosphonates, and IL-15, both with IL-2 and alone, has been described to contribute to reduced apoptosis and higher cytokine and cytotoxic mediator expression upon restimulation [18]. However, *in vitro* expansion of UCB *γδ* T cells with the bisphosphonate alendronate or zoledronate and a low dose of IL-2 has been described to preferentially induce differentiation into a cytokine production rather than a cytotoxic phenotype [15].

The development of *γδ* T cell products for use after hematopoietic stem cell transplantation (HSCT) is an attractive prospect. The clinical significance of *γδ* T cells in the HSCT context is clearly demonstrated in reports showing that higher frequencies of *γδ* T cells after transplantation are associated with favorable outcome [29–31]. Importantly, reconstitution of *γδ* T cells after HSCT depends mainly on the graft source, with poor reconstitution of *γδ* T cells seen after umbilical cord blood transplantation (UCBT). The impact of graft source on *γδ* T cell reconstitution can most likely be attributed to the number and quality of the *γ*δ T cells present in the graft [31], stressing the potential of *γδ* T cell immunotherapy in UCBT recipients, preferentially with graft-derived UCB *γδ* T cells.

The aim of the present study was to further explore the in vitro culture of UCB *γδ* T as a potential source of cells for adoptive cell therapy (ACT), with specific focus on treatment after UCBT. The first step towards the development of a successful ACT strategy is the establishment of an efficient production protocol easily conformable to good manufacturing practice (GMP) regulations. We have here initialized the development of such a protocol, using the experiences of others, and we are continuing to explore optimal production conditions. The choice of the reagents for the protocol, zoledronate, and IL-2 was based on their availability in formulations conforming to GMP standards. We also chose to focus on the expansion of *δ*2*γ*9 T cells, a subset known to be easily and reliably expanded *in vitro*, and to have antitumoral and anti-infectious properties, making it suited for use as adoptive therapy after HSCT. We found that we could successfully expand products with a high percentage of *γδ* T cells present, of which the majority were *δ*2*γ*9 T cells in spite of very small numbers of this subset at baseline. We also found that they displayed both cytokine production and cytotoxic antitumor capacity. We see considerable potential in this protocol, especially in the context of UCBT, and hope to further develop this culture protocol towards clinical application.

## 2. Material and Methods

### 2.1. Umbilical Cord Blood Units

Umbilical cord blood (UCB) was collected from healthy volunteers giving birth at the maternity ward at Karolinska University Hospital in Huddinge, Stockholm, Sweden, for the Swedish Umbilical Cord Blood Bank, and was made available for research due to insufficient cell numbers for clinical use. Collected UCB units were received without any labeling that could enable tracking of the donor and were given unique numbers according to order of collection. The UCB donors were three females and five males. Peripheral blood mononuclear cells (PBMC) were collected from healthy volunteers, two females and one male. All donors had given their informed consent prior to donation in accordance with the guidelines and regulations stipulated by the Karolinska Institute and with the declaration of Helsinki. The project was approved by the regional ethical board (2007/4:10).

### 2.2. Sample Preparation and Cell Culture

Mononuclear UCB and PB cells were obtained by density gradient separation (Lymphoprep, Fresenius Kabi Norge AS) and washed and cryopreserved in complete medium (as defined below) supplemented 10% di-methyl sulphoxide (DMSO, Wak-Chemie Medical GmbH, Steinbach, Germany) in liquid nitrogen. The frozen samples were thawed and washed prior to culture, and for all but the preliminary experiment, a depletion of CD56^+^ cells was performed for all UCB cultures using a 2-step positive selection method by first staining the cells with CD56-APC antibody (BD Bioscience, Franklin Lakes, NJ, USA) and then adding MACS anti-APC beads (Miltenyi, Bergisch Gladbach, Germany) according to manufacturers' instructions. Cells were then seeded at a concentration of 1 × 10^6^ viable mononuclear cells/ml in complete medium, defined as 1640 RPMI (Life Technologies (Gibco)) supplemented with 10% pooled human AB-serum (Department of Transfusion Medicine at Karolinska University Hospital Huddinge), 100 IU/ml penicillin G, 100 mg/ml streptomycin, 0.25 mg/ml amphotericin B (Life Technologies (Gibco)), and 2 mM L-glutamine (Sigma Aldrich Inc, St Louis, MO). Recombinant IL-2 and the bisphosphonate zoledronate were added in varying concentrations (0–500 IU/ml and 0–10 *μ*M, resp.) according to Table 1. Cell culture was performed at 37°C at 5% CO_2_. Viable cells were counted using trypan blue exclusion every other day and replated to maintain cell concentration. Cells were replated using a medium containing IL-2 according to protocol, but zoledronate was only added on day 0. On day 7, cells were spun down by centrifugation, and the supernatant was removed in order to completely replace the culture medium. On day 14, cells were counted, harvested, and cryopreserved in complete medium supplemented with 10% DMSO.

### 2.3. Flow Cytometry

Cell surface staining was performed as described previously [32]. Briefly, cells were incubated at 4°C for 20 min with antibodies diluted in PBS supplemented with 1% FCS (staining buffer) and washed before FACS analysis. Acquisition was performed on a FACSCANTO flow cytometry instrument (BD) using the FACSDiva software (BD). The acquired data was analyzed with FlowJo (Tree Star Inc., Ashland, OR) and subsequently using the bh-SNE algorithm in the CYT software (Dana Pe'er Lab, ref) running on MatLab (MathWorks Inc., Natick, MA, USA). Control stained samples were used for gating according to the fluorescence minus one technique. The antibodies used in the flow cytometry panels are described in Table 1.

### 2.4. Spectratyping

The *γδ* TCR repertoire with regard to the main *δ* families (V*δ*1, V*δ*2, and V*δ*3) and *γ* families (V*γ*9, V*γ*10, and V*γ*11) was assessed using the spectratyping method adapted from Rådestad et al. [33]. Briefly, DNA was extracted from expanded UCB cells, and amplifications of 12 *δ* chain subfamilies and 9 *γ* chain subfamilies were performed by multiplex PCR reaction using primers as described before [34] (Tables S1–3). The PCR was performed using AmpliTaq Gold 360 Master Mix (Applied Biosystems), specific primers in a final concentration of 200 or 400 nanoMoles as indicated in Table S1 and S2, and 100 ng of DNA, with a thermal cycler PCR machine (PTC-200, MJ Research, Watertown, MA). The process included the following steps: initial denaturation at 95°C for 10 minutes, followed by 35 cycles each of 94°C for 30 sec, 60°C for 45 sec, 72°C for 60 sec, and a final elongation step at 72°C for 10 minutes; and capillary electrophoresis where each PCR product was mixed with formamid (FA, HiDi Formamide) and size standard (GeneScan 400HD Rox Size std, Applied Biosystems) in 96-well MicroAmp plates (Applied Biosystems). Samples were analyzed using 3130 × 1 Genetic Analyser (Applied Biosystems). The results were analyzed using the PeakScanner software (Applied Biosystems).

### 2.5. Cytokine Production Assay

Relative expression of cytokine genes was determined for expanded UCB and PB *γδ* T cells cultured with 5 *μ*M zoledronate and 200 IU IL-2/ml medium. Briefly, cryopreserved cells were thawed and RNA was extracted from sorted *γδ* T cells (TCR *γ*/*δ* T cell isolation kit, Miltenyi) using the PureLink™ RNA Mini Kit (Invitrogen, ThermoFisher) and converted to cDNA using SuperScript™ IV VILO™ Master Mix (ThermoFisher) according to manufacturer's instructions. Real-time (Rt) PCR was performed on 7500 fast real-time PCR instrument (Applied Biosystems), using TaqMan Gene Expression Assays for *IL-1B*, *IL-2*, *IL-6*, *IL-7*, *IL-8*, *IL-12B*, *IL-15*, and *IL-17* genes and human ACTB gene as reference gene.

### 2.6. Cytotoxicity Assay

*γδ* killing was assessed using a cytometry-based assay as previously described [35]. Briefly, cryopreserved expanded UCB and PB *γδ* T cells cultured with 5 *μ*M zoledronate and 200 IU IL-2/ml medium were thawed and cocultured with target tumor cells from the human cholangiocarcinoma cancer cell line Hucct-1. Hucct-1 cells were labeled with the CellTrace™ Violet Cell Proliferation marker (CTV, Thermo Fisher) for easy identification and incubated with cultured *γδ* cells at 37°C, with 5% CO_2_ for 24 hours at an effector: target ratio of 10 : 1. Cell viability was then assessed by flow cytometry in CTV positive cells using annexin V. The % of target cells killed by *γ*δ T cells was calculated by the following formula: % *γδ* T cell killing = 100 − (*% viable CTV + Hucct-1 cells in cocultures with γδ cells)/(% viable CTV + Hucct-1 cells in control culture without γδ cells)* × 100.

### 2.7. Statistics

Data was analyzed and displayed using Prism 6 (GraphPad, San Diego, CA). The T cells cultured under different conditions were paired according to level of IL-2 and the presence or absence of zoledronate in the culture media. Due to the limited sample size, nonparametric pair-wise comparisons were performed using the Wilcoxon rank-sum test, and in the instances where 5 or fewer measurements were available in either group, the Mann–Whitney *U* test.

The marker expression patterns of cells cultured in different culture conditions were also compared using the bh-SNE algorithm, which, briefly, converts multidimensional data into bidimensional, mapping similarities by first calculating a pairwise distance matrix for the high-dimensional space and transforming it into a similarity matrix using a varying Gaussian kernel. A random bidimensional map is then rendered, and pairwise similarities are calculated for the low-dimensional space created. The map is then optimized in iteration steps where the calculated similarity between any given two cells is rechecked to optimally redistribute them on the map. The analysis was performed using the CYT software from Dana Pe'ers lab on the Matlab platform [36]. Here, flow cytometry data was gated to include only CD3^+^ single-cell events and exported as separate files using FlowJo. The fluorescence data was arcsin transformed with a cofactor of 150 in CYT for comparability, and subsamples of 5000 events were obtained randomly from the sample files before bh-SNE analysis.

The analysis of viability and apoptosis was performed using the SPICE software [37], which generated graphics illustrating the differences in the proportion of dead and apoptotic cells between the groups, and the statistical analysis was performed using the built-in test in the software, applying 1,000,000 permutations, as described previously [37].

## 3. Results

### 3.1. The Proliferation of UCB *γδ* T Cells Is Influenced by the Concentrations of IL-2 and Zoleronate in the Culture Medium

UCB units were divided into five parts that were cultured in complete medium containing 50 IU IL-2/ml alone or, 50, 100, 200, or 500 IU IL-2/ml combined with 5 *μ*M zoledronate. The selection of the experimental conditions was based on an initial experiment where a UCB unit was divided into 13 parts cultured separately with different concentrations of IL-2 (50, 100, 200, 400, or 600 IU/ml culture medium) and zoledronate (5 *μ*M or 10 *μ*M), or in complete medium only. Assessment on day +14 for viable cell count using trypan blue exclusion and for phenotype with flow cytometry indicated that the lower dose of zoledronate (5 *μ*M) resulted in the best results with regard to viability, proliferation, and percentage of *γδ* T cells. Zoledronate in the absence of IL-2 had limited effect on the proliferation of *γδ* T cells. IL-2 was found to reinforce the zoledronate-induced proliferation of *γδ* T cells already from a low concentration (data not shown). A sizeable number of CD56^+^ CD3^−^ NK cells was seen at the end of expansion, which led us to introduce bead-based depletion of CD56^+^ cells into the protocol. After the first experiments indicated that 500 IU IL-2/ml had limited, if any, additional effect on *γδ* T cell proliferation compared to 200 IU IL-2/ml, the final cell cultures were performed with either 50, 100, or 200 IU IL-2/ml and 5 *μ*M zoledronate. Lastly, for comparison purposes, peripheral blood mononuclear cells (PBMC) from healthy volunteers were cultured in medium with 200 IU IL-2/ml and 5 *μ*M zoledronate.

**Figure 1:**
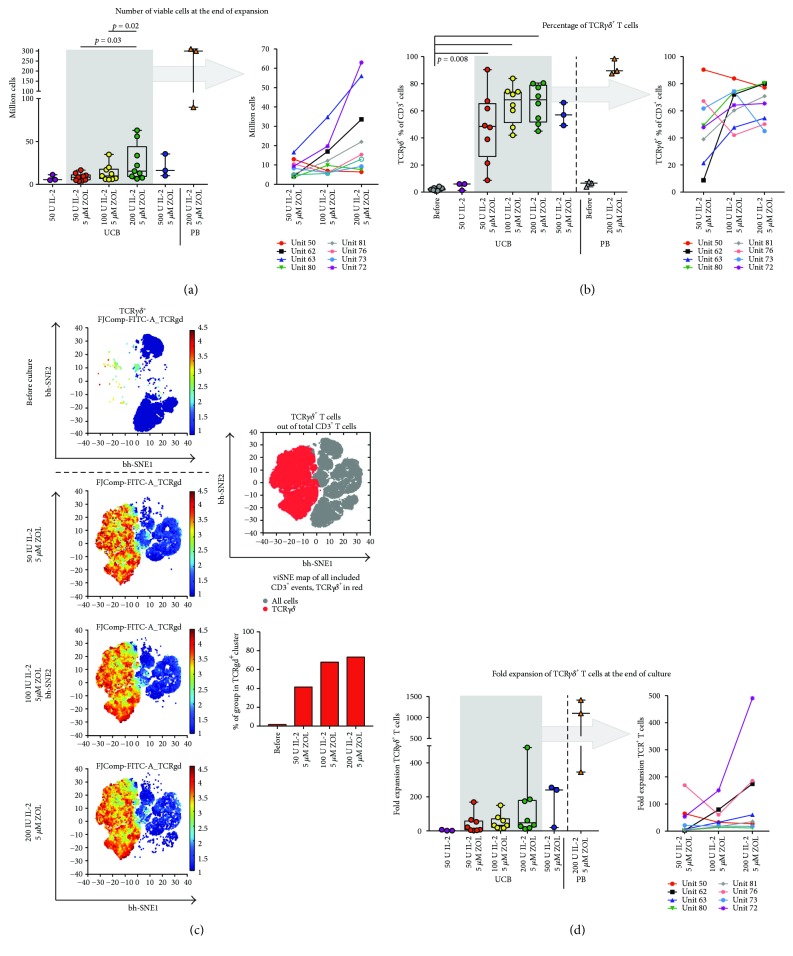
Expansion of *γδ* T cells. The number of viable cells after 14 days of culture is displayed according to culture conditions in (a). Here, and in (c) and (d), the grey box in the left graph encompasses the results of UCB *γδ* T cells cultured with 5 *μ*M zoledronate and 50, 100, or 200 IU IL-2/ml medium, and in the in right graph, indicated by the arrow, the data inside the box is displayed separately for each cultured UB unit (each unit is indicated by a differently colored line). In (b) and (c), the proportion of *γδ* T cells is visualized in two different manners. In (b), the percentage of TCR*γδ* ^+^ T cells is shown before and after culture. In (c), the same data is displayed for expansions of UCB cells using the bh-SNE algorithm to analyze and visualize the data. The panels to the left display the fluorescence intensity in the flow cytometry channel for TCR*γδ*, normalized using arcsin transformation with cofactor 150, for every cell in the displayed groups. In the top right panel, an overview of the cells in all samples is displayed and gated according to where the TCR*γδ* ^+^ cells are clustered. The lower right panel shows the percentage of cells in each group found in the TCR*γδ* ^+^ cell cluster. The fold expansion of *γδ* T cells is displayed in (d). IL-2: interleukin 2; TCR: T cell receptor; ZOL: zoledronate.

Assessment of viable cell counts, using trypan blue exclusion, and the phenotype of the cultured cells was performed after 14 days of culture. The results, displayed in Figure 1, indicated that the highest number of viable UCB cells was obtained using medium containing zoledronate and 200 IU IL-2/ml (Figure 1(a)). However, the cell counts were comparatively lower in cultured UCB cells than in PBMC expansions, as might be expected based on the small numbers of *γ*9*δ*2 T cells in baseline UCB. The percentage of TCR*γδ* UCB T cells was high after culture with medium containing zoledronate with IL-2 concentrations between 50 and 200 IU/ml, with a tendency towards higher percentages *γδ* T cells with higher IL-2 concentrations (Figures 1(b) and 1(c)). The percentage of *γδ* T cells in PBMC cultures tended to be slightly higher (Figure 1(b)). The change from a T cell population with a small proportion of *γδ* T cells in UCB unit at baseline sample to a postculture T cell population containing a majority of *γδ* T cells is illustrated in Figure 1(c), where graphical “maps” generated using the bh-SNE algorithm are displayed. The analysis was performed for UCB cultures on gated CD3^+^ T cells. The total proportion of *γδ* T cells in each group (all included samples assessed together) indicates that, on a group basis, the highest proportion of *γδ* T cells was achieved using 100–200 IU IL-2/ml medium (Figure 1(c), lower right panel).

The fold expansion of *γδ* T cells was robust, with median fold changes of 14, 33, and 47 in UCB expansions cultured with medium containing zoledronate and 50, 100, and 200 IU IL-2/ml, respectively (Figure 1(d)). The fold change of PB cultures was higher, with a median of 1099.

There seemed to be no discernable difference with regard to percentage of *γδ* T cells at baseline (Supplementary Figure 1a) or after culture, or with regard to fold expansion at the end of culture (data not shown) between UCB cultures derived from male newborns compared to ones from female newborns.

### 3.2. Superior Viability of Cultured UCB *γδ* T Cells Is Achieved Using Medium Level Concentrations of IL-2

**Figure 2:**
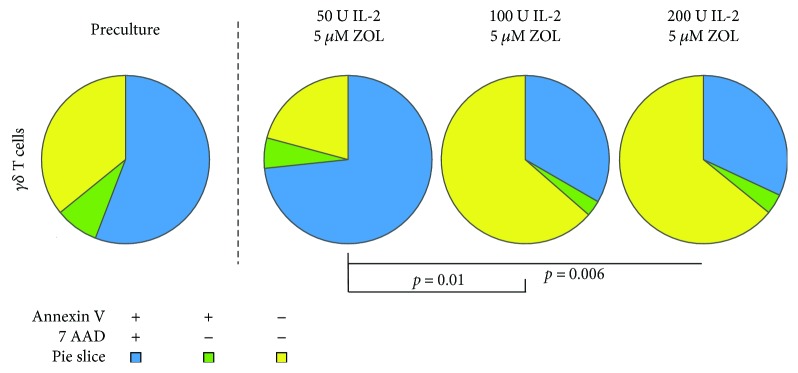
Cell death and apoptosis. Viability and apoptosis. The mean proportion of viable (annexin V^−^ 7AAD^−^) cells, apoptotic (annexin V^+^ 7AAD^−^) cells, and dead (annexin V^+^ 7AAD^+^) cells are displayed and analyzed using the SPICE software. Statistical comparisons were performed using the built-in statistical analysis in the SPICE software. IL-2: interleukin 2; ZOL: zoledronate.

We assessed cell death and apoptosis in the UCB cells before and at the end of culture by flow cytometry using a combination of the markers 7-AAD (7-Aminoactinomycin D) and annexin V. Cells positive for both these markers were defined as dead or dying, and cells single-positive for annexin V were defined as apoptotic, as has been described before [38]. Cells negative for both these markers were defined as viable. We found that the proportion of viable, apoptotic and dead *γδ* T cells, differed significantly after culture with 5 *μ*M zoledronate and 100–200 IU IL-2/ml compared with 5 *μ*M zoledronate and 50 IU IL-2/ml, with a higher proportion of viable cells and less dead and apoptotic cells with the former (Figure 2).

### 3.3. The Majority of Cultured UCB *γδ* T Cells Express TCR V*δ*2 and TCR V*γ*9 and Have a Memory Phenotype

We further wanted to establish which subtype of *γδ* T cells was generated by our expansion protocol, based on which TCR *γ* chains and *δ* chains made up the TCR of the cultured *γδ* T cells. Due to technical factors regarding the flow cytometry panel setup, the analysis of pan-TCR*γδ* and V*γ*9, V*δ*1 and V*δ*2, respectively, in the same panel was not possible. However, using the known percentage *γδ* T cells/CD3^+^ T cells and comparing it with the percentage V*γ*9^+^, V*δ*1^+^, and V*δ*2^+^/CD3^+^ T cells, the proportions of *γ*9, *δ*1, and *δ*2 *γδ* T cells could be estimated.

**Figure 3:**
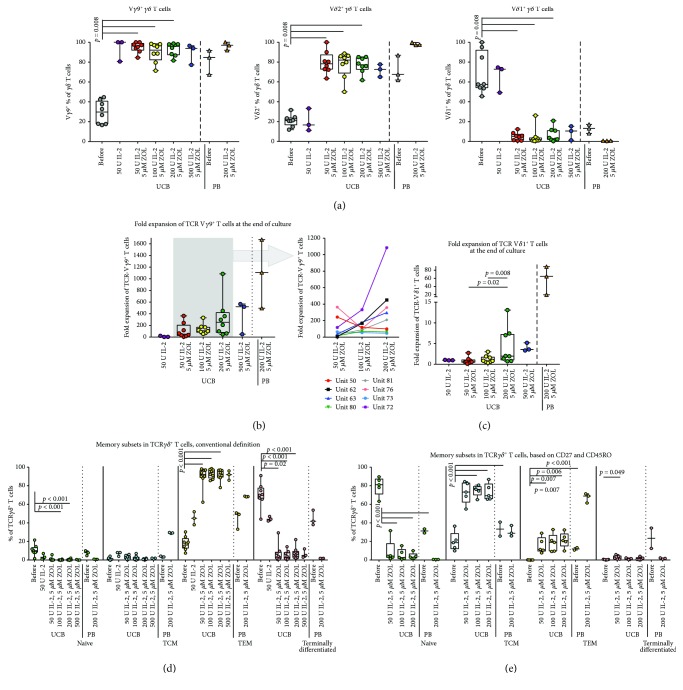
*γδ* chain usage and memory phenotype in cultured *γδ* T cells. Phenotype of the cultured *γδ* T cells. The phenotype of the *γδ* T cells with regard to the expression of memory markers and specific *γ* and *δ* chains was assessed by flow cytometry. In (a), the percentage of *γδ* T cells positive for V*γ*9 (left panel), V*δ*2 (middle panel), and V*δ*1 (right panel) is shown. The fold expansion of V*γ*9^+^ T cells and V*δ*1^+^ T cells is displayed in (b) and (c), respectively. In (b), the grey box in the left graph contains the UCB *γδ* T cells cultured with 5 *μ*M zoledronate and 50, 100, or 200 IU IL-2/ml medium, and in the right graph, indicated by the arrow, the data inside the box is displayed separately for each cultured UB unit (each unit is indicated by a differently colored line). The memory phenotype of the *γδ* T cells before and after culture, using a definition based on the expression of CD45RO and CCR7, is displayed in (d). Naïve T cells were defined as CD45RO^−^ CCR7^+^, central memory as CD45RO^+^ CCR7^+^, effector memory as CD45RO^+^ CCR7^−^, and terminally differentiated cells were defined as CD45RO^−^ CCR7^−^. The memory phenotype, defined by the expression of CD45RO and CD27, is displayed in (e). Naïve T cells were defined as CD45RO^−^ CD27^+^, central memory T cells as CD45RO^+^ CD27^+^, effector memory T cells as CD45RO^+^ CD27^−^, and terminally differentiated T cells as CD45RO^−^ CD27^−^. IL-2: interleukin 2; TCM: central memory T cells; TCR: T cell receptor; TEM: effector memory T cells; ZOL: zoledronate.

As expected, a majority expressed V*γ*9 and V*δ*2, respectively, in both UCB and PBMC expansions, while the percentage of V*δ*1^+^ cells was reduced from baseline (Figure 3(a)). Interestingly, we noticed that a slightly higher percentage of cells was consistently found to be positive for V*γ*9 than for V*δ*2 in UCB-derived cultures but not PBMC-derived cultures. At baseline, there was a nonsignificant tendency towards higher proportions of V*δ*1^+^ T cells in UCB units from male newborns than in units from females (Supplementary Figure 1b). There were no discernable differences for the other subsets at baseline (Supplementary Figure 1b) or after culture (data not shown).

The fold expansion of *γ*9 T cells was impressive in both PB and UCB cultures. The small numbers of cells found in UCB at baseline expanded dramatically, with median fold changes of 50, 110, and 253 in the expansions cultured with medium-containing zoledronate and 50, 100, and 200 IU IL-2/ml, respectively, and with a maximum fold change of 1085 in a culture with 200 IU IL-2/ml and 5 *μ*M zoledronate (Figure 3(b)). The PB *γ*9 T cells had a median fold change of 1109. In contrast, the median fold expansion for V*δ*1^+^ T cells was moderate (Figure 3(c)). No visible difference in fold expansion could be seen between male and female UCB units.

We also studied the memory phenotype, using a standard definition based on the coexpression of CD45RO and CCR7, originally defined for conventional PB *αβ* T cells. Naïve T cells are CD45RO^−^ CCR7^+^, central memory is CD45RO^+^ CCR7^+^, effector memory is CD45RO^+^ CCR7^−^, and terminally differentiated cells are defined as CD45RO^−^ CCR7^−^. We found that the predominant phenotype after culture corresponded with the effector memory phenotype (CD45RO^+^ CCR7) in both UCB and PBMC expansions. However, the baseline UCB and PB *γδ* T cell population had a high percentage of terminally differentiated cells according to this definition (Figure 3(d)). In line with several previously published articles, we then analyzed the memory phenotype using a definition where CCR7 is substituted with CD27 [15, 18, 39, 40]. According to this definition, the majority of the baseline UCB *γδ* T cells was naïve (CD45RO^−^ CD27^+^), with some representation of central memory (CD45RO^+^ CD27^+^) and effector memory (CD45RO^+^ CD27^−^) cells, and almost no terminally differentiated cells (CD45RO^−^ CD27^−^, Figure 3(e)). Interestingly, PB *γδ* T cells were divided almost evenly between the memory subsets at baseline, with slight predominance of the naïve and central memory phenotypes. In the cultured UCB cells, the largest subset was positive for CD45RO but had maintained CD27 expression, classifying them as antigen-experienced cells with a central memory phenotype, while the largest subset in the PBMC expansions had upregulated CD45RO but lost CD27 expression, indicating an effector memory phenotype with this definition.

### 3.4. Spectratyping Showed a Polyclonal Pattern, Indicating a Postexpansion Cell Product Containing a Variety of TCR *γδ* Specificities

Spectratyping analysis was performed in order to assess the clonality of the repertoire of the expanded UCB *γδ* T cells. The subfamilies of *γ* and *δ* chains can be classified as polyclonal (here defined as >6 peaks), oligoclonal (defined as 3–6 peaks), or monoclonal (defined as <3 peaks).

**Figure 4:**
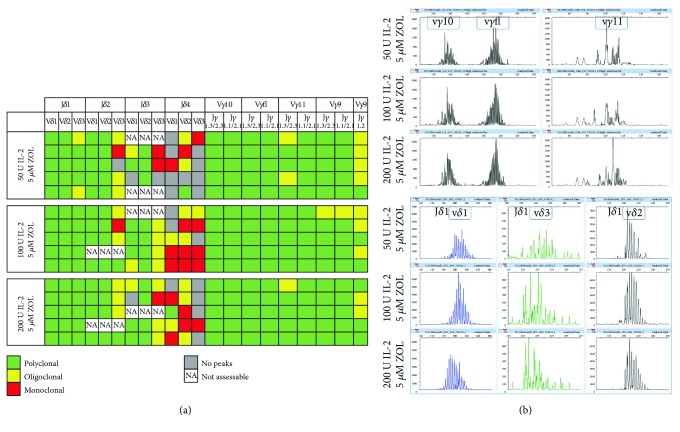
Spectratyping results. TCR repertoire of the cultured *γδ* T cells. Spectratyping results with the expression of *γ* and *δ* chains in the TCR of the expanded *γδ* T cells are displayed, with regard to the clonality of each subfamily, for the postculture sample, in (a). Each row displays the results for one cultured umbilical cord blood unit. Polyclonal populations were defined as having >6 peaks (green squares), oligoclonal populations had 3–6 peaks (yellow squares), and monoclonal populations were defined as having <3 peaks (red squares). Grey squares indicate that no peaks were detected for that subfamily. NA, an abbreviation of not assessable, indicates that data could not be obtained due to technical reasons, such as insufficient DNA material in the test. In (b), representative examples of data analyzed with the peak scanner software is displayed for the V*γ*10 J*γ*1.3/2.3, V*γ*fl J*γ*1.3/2.3, V*γ*11 J*γ*1.3/2.3, and V*δ*1 J*δ*1, V*δ*3 J*δ*1, and V*δ*2 J*δ*1 subfamilies.IL-2: interleukin 2; ZOL: zoledronate.

The results indicated that the expansion protocol resulted in similar TCR *γδ* repertoires in all tested culture conditions (5 *μ*M zoledronate combined with either 50, 100, or 200 IU IL-2/ml culture medium). The cultured cell populations had mainly polyclonal repertoires in the V*δ*1-J*δ*1, V*δ*2-J*δ*1, and V*δ*3-J*δ*1 subfamilies, the V*δ*1-J*δ*2 and V*δ*2-J*δ*2 subfamilies, the V*δ*1-J*δ*3 and V*δ*2-J*δ*2 subfamilies, and for the majority of the V*γ*9, V*γ*10, V*γ*11, and V*γ*fl subfamilies (Figure 4(a)). This indicates that the culture procedure caused independent expansion of a multiplicity of different clones, leading to a final cell population with a broad TCR repertoire.

Several subfamilies had a predominantly oligoclonal or monoclonal repertoire or were missing peaks in a several analyzed samples, however. These included the V*δ*3-J*δ*2 subfamily and all the V*δ*1-J*δ*4, V*δ*2-J*δ*4, and V*δ*3-J*δ*2 subfamilies. Samples with missing peaks in the V*δ*1-J*δ*4 and V*δ*3-J*δ*4 subfamilies were slightly more common in the cultures exposed to 50 IU IL-2/ml culture medium (4/5, in both) than in the expansions cultured with 100 or 200 IU IL-2/ml (2/5 and 1/5, respectively, and 1/5 and 4/5, respectively, Figure 4).

### 3.5. Cytokine Production Differed between Cultured Umbilical Cord Blood and Peripheral Blood *γδ* T Cells

**Figure 5:**
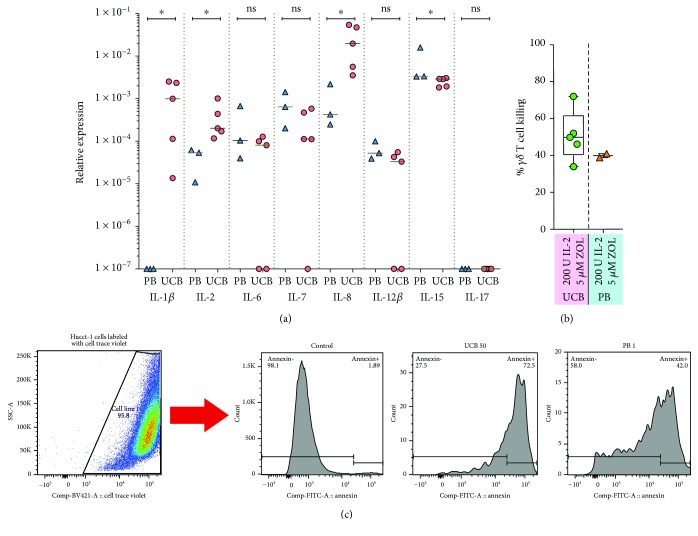
*γδ* T cell cytokine production and cytotoxicity. The results of real-time PCR analysis of the gene expression for the cytokines IL-1, IL-2, IL-6, IL-7, IL-8, IL-12, IL-15, and IL-17 in UCB and PB *γδ* T cells cultured with 5 *μ*M zoledronate and 200 IU IL-2/ml medium displayed in (a). In (b) and (c), the results of a cytotoxicity assay based on coculture of Celltrace Violet^®^- (CTV-) labeled cholangiocarcinoma cells from the Hucct-1 cell line and thawed UCB and PB *γδ* T cells previously cryopreserved after culture with 5 *μ*M zoledronate and 200 IU IL-2/ml medium. (b) displays the results for all tested units, and (c) shows representative FACS plots of the gating strategy for CTV-labeled tumor cells (left) and histogram plots of annexin V^+^ gated cells from a control culture (no *γδ* T cells added), and from cocultures with UCB and PB *γδ* T cells, respectively (three plots to the right). ^*∗*^ *p* < 0.05.

Cytokine gene expression was measured by Rt-PCR in expanded UCB and PBMC *γδ* T cells cultured with 5 *μ*M zoledronate and 200 IU IL-2/ml medium (selected based on superior fold expansion of UCB *γδ* T cells, especially of *γ*9 T cells). IL-1*β*, IL-2, IL-6, IL-12*β*, IL-15, and IL-17 were examined, and, interestingly, the expression of IL-1*β*, IL-2, and IL-8 was significantly higher in UCB *γδ* T cells (Figure 5(a)). This was especially striking in the case of IL-1*β*, as the PB *γδ* T cells expressed IL-1*β* at a very small extent. The gene expression with regard to IL-17 was low in both UCB and PB *γδ* T cells (Figure 5(a)).

### 3.6. The Cultured UCB *γδ* T Cells Display Cytotoxic Capacity

A cytotoxicity assay using coculture of *γδ* T cells and CTV-labeled cells from the Hucct-1 tumor cell line was performed to test the killing capacity of the cultured *γδ* T cells. We selected expansions that had been cultured with 5 *μ*M zoledronate and 200 IU IL-2/ml medium for this assay. Target cell killing, measured as percentage annexin V positivity in tumor cells, ranged from 33.9–72%, with a median of 49.8% in the 5 tested UCB culture products (Figure 5(b)). The two PBMC cultures tested for comparison showed killing rates of 38.8 and 40.9 (Figure 5(b)).

### 3.7. The Expression of Phenotypical T Cell Markers Is Affected by the Culture Process

**Figure 6:**
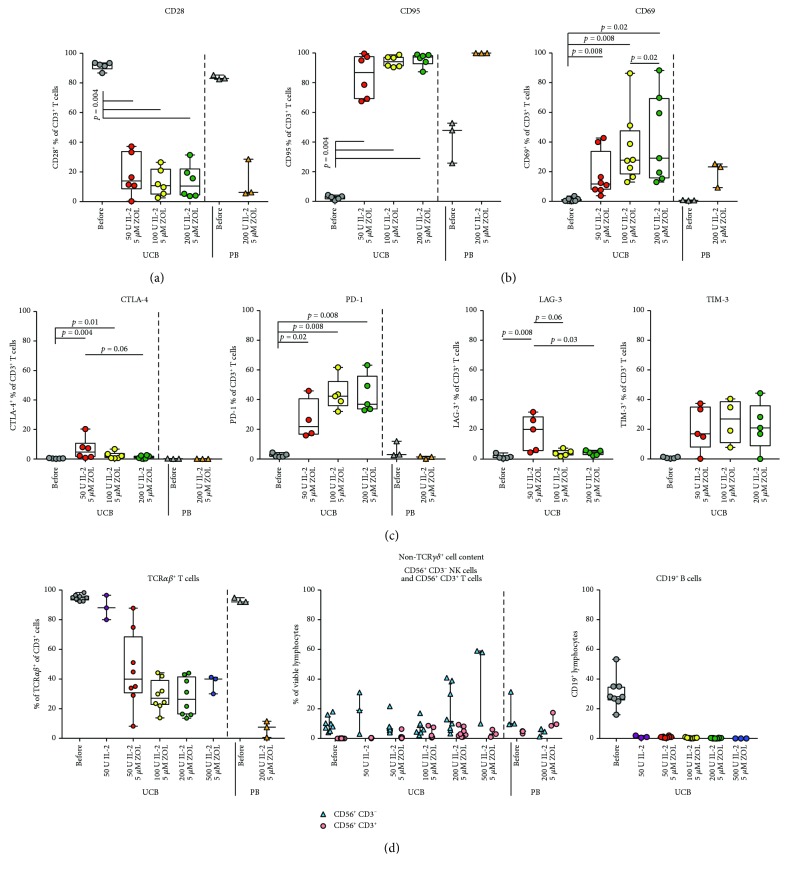
Marker expression in total T cells. Cosignaling marker expression. The phenotype of the cultured T cells from UCB and PB with regard to the expression of markers of activation and costimulatory and inhibitory receptors was assessed by flow cytometry. The percentage of cells expressing the costimulatory marker CD28 (a), the activation markers CD95 and CD69 (b), and the coinhibitory markers CTLA-4, PD-1, LAG-3, and TIM-3 (c) are displayed. In (d), the percentage of TCR*αβ* ^+^ T cells before and after expansion is displayed in the left panel. The percentages of CD56^+^ CD3^−^ and CD56^+^ CD3^+^/viable cells are displayed in the middle panel of (d), and the right panel shows the percentage of CD19^+^ lymphocytes before and after culture. IL-2: interleukin 2; TCR: T cell receptor; ZOL: zoledronate.

We wanted to further elucidate the kinetics of activation markers and cosignaling receptors on the cultured cells and thus analyze the costimulation marker CD28, the activation marker CD69, the activation/proapoptosis marker CD95, and the coinhibitory receptors CTLA-4, PD-1, LAG-3, and TIM-3. Due to technical considerations in the setup of flow cytometry panels, these receptors were assessed on total CD3^+^ T cells. We could see that significant downregulation of CD28 and upregulation of CD95 and CD69 was induced during the culture process compared to the baseline values in both UCB- and PBMC-derived culture products (Figures 6(a) and 6(b)). There was a significantly higher proportion of CD69^+^ UCB T cells in expansions cultured in medium with 200 IU IL-2/ml compared to with 50 IU IL-2 /ml, with a nonsignificant trend towards the same for CD95 (Figure 6(b)). The expression of co-inhibitory receptors was also affected: PD-1 showed a general increase in expression in UCB but not PB *γδ* T cells after culture. For TIM-3, there was a nonsignificant trend toward the same (only studied in UCB expansions). The percentage of CTLA-4^+^, in UCB and PB cultures, LAG-3^+^ (only studied in UCB cultures), and T cells was significantly increased preferentially in UCB T cells cultured with 50 IU IL-2/ml compared to baseline and to UCB T cells cultured with medium containing 200 IU IL-2/ml (Figure 6(c)).

### 3.8. The Representation of Other Lymphocyte Subsets in the Final Culture Is Dominated by CD56^+^ NK^−^ and NKT Cells

Even the UCB and PB cultures with a predominant *γδ* T cell population had a remaining proportion of other lymphocytes, containing a mixture of *αβ* T cells and NK cells (Figure 6(d), left and middle panel). We also noticed a percentage of CD56^+^ CD3^+^ cells (Figure 6(d), middle panel). The remaining percentage of TCRα*β* ^+^ T cells was significantly reduced in cultures exposed to zoledronate and IL-2 in a manner corresponding to the increased percentage of TCR*γδ* ^+^ T cells (Figure 6(d), left panel). At baseline, a nonsignificant trend towards higher percentages of NK cells was seen in UCB units from male newborns compared to female donors (Supplementary Figure 1a).

No B-cells could be detected in the cultures on day +14 (Figure 6(d), right panel, only tested for UCB cultures).

## 4. Discussion

*In vitro* expansion of UCB *γδ* T cells is a challenge. This is due to several factors, such as the scarcity of *γ*δ T cells in UCB [15] and the very low frequency of the *γδ* T cell subset known to be easily expanded *in vitro*: the *γ*9*δ*2^+^ T cells [18, 19]. Also, the functional immaturity of UCB *γδ* T cells [15], including a reduced capacity to respond to IL-2 [20, 21], and the previous observations that indicate a tendency towards development of cytokine production rather than cytotoxicity during *in vitro* culture [15] contribute to the difficulties. This is especially true for the development of a culture protocol robust and reproducible enough for the production of adoptive cell therapies for clinical use, as the goal here is to manufacture cell products that are consistent in quality and that contain the desired cellular phenotype with antitumoral and anti-infectious properties. This study was performed with the aim of exploring the possibility of translating the protocols used for expansion of peripheral blood *γδ* T cells to the UCB setting, with the long-term goal to explore the possibility of adoptive *γδ* T cell therapy after UCBT. *γδ* T cell reconstitution is known to be important for HSCT outcome [29–31], perhaps due to their MHC-unrestricted antigen recognition and antitumoral and anti-infectious properties. The reconstitution of *γδ* T cells after UCBT is especially poor, and thus there is a need for innovative strategies, such as expanded *γδ* T cells from an aliquot of the original graft. We see the results presented here as a stepping-stone toward the development of such therapies.

We based the choice of zoledronate and IL-2 for the culture protocol on their easy availability in GMP-compliant preparations as clinically approved drugs and on the extensive published experience showing that they can be used to reliably expand *γ*9*δ*2 T cells. The choice of zoledronate was made based on results showing better proliferative response in UCB *γδ* T cells to zoledronate compared to pamidronate [15]. We did consider IL-15, as this cytokine has been described to confer less apoptotic *γδ* T cells after culture, and higher cytokine and cytotoxic mediator expression upon restimulation [18]. However, the final choice of IL-2 was based on this cytokine having, in addition to easy availability in GMP-preparations, the largest documented experience of *in vitro* as well as *in vivo* use.

We found that the protocol here used, combining zoledronate and IL-2 at different concentrations, led to a substantial proliferation of UCB *γδ* T cells. The resulting culture contained a majority of CD3^+^ T cells, out of which a varying proportion expressed a *γδ* TCR. The proportion of *γδ* T cells in UCB cultures seemed to increase with the concentration of IL-2 in the culture medium up to 200 IU/ml and the combination of 5 *μ*M zoledronate, and 100–200 IU IL-2/ml resulted in the highest percentages of UCB *γδ* T cells (Figure 1(b)). No significant differences could be noted in fold expansion of *γδ* T cells or the percentage of *γδ* TCR^+^ in UCB cultures treated with 100 IU or 200 IU IL-2/ml. The only difference was that the highest IL-2 concentrations were associated with a tendency towards more CD56^+^ NK cells at the end of the culture (Figure 6(d)). Another important aspect was that a concentration of IL-2 of 100–200 conferred a significantly higher percentage of viable UCB cells at the end of culture than a concentration of 50 IU IL-2/ml (Figure 2).

The majority of the cultured *γδ* T cells expressed V*γ*9, and to a slightly lesser extent, V*δ*2. This was not unexpected, as *γ*δ T cells with a TCR made up by a *γ*9 chain and a *δ*2 chain are known to be activated *in vitro* by the presence of bisphosphonates, such as zoledronate, in the culture medium [4, 6, 22, 23, 28]. Two interesting aspects of this finding are worthy of notice, however. The first is the very substantial fold expansion of *γ*9 T cells. The median fold expansion and maximum fold expansion of subset with the expansion conditions with the highest yield, treated with 5 *μ*M zoledronate and 200 IU IL-2/ml medium, were 253 and 1085, respectively (Figure 3(b)). Compared to PB cultures treated with 5 *μ*M zoledronate and 200 IU IL-2/ml, the fold expansion of total *γδ* T cells was considerably lower in UCB cultures, but the difference with regard to *γ*9 T cell expansion was noticeably less (Figures 1(d) and 3(b)). This indicates that UCB *γ*9 T cells have an impressive proliferation potential [13, 14]. This is a prerequisite for the development of potential cell therapy schemes using bisphosphonates, as the percentage of this subset is small in baseline UCB.

The other interesting feature seen in the UCB cultures, but not in the PBMC-derived expansions, is the discrepancy in the percentages of positive cells between V*γ*9 and V*δ*2, with a somewhat smaller proportion of the cultured *γδ* T cells staining positive for V*δ*2. This could indicate that the cultured cell population contains a smaller subset of V*γ*9^+^ cells with TCRs made up with another V*δ* chain, such as *δ*1. The TCRs of V*δ*1^+^ T cells are known to include various V*γ* chains [41–43], and thus the low proportion of V*δ*1^+^ T cells found to persist after culture could indicate that a population of *γ*9*δ*1 T cells is maintained through the culture process. UCB contains higher levels of this cell subset [18, 44]. High frequencies of *γ*9*δ*1 T cells have been described in a patient with a recurrent fever syndrome [45], indicating that this subset can be induced to extensive proliferation in specific situations. The ligands recognized by V*δ*1 T cells (with different V*γ* chains) remain largely uncharacterized but include CD1 family proteins [9, 43] and MICA/B through activation via the TCR and the NK cell receptor NKG2D [10, 11]. MICA and MICB expression has been described in a variety of tumors and has been associated with an increased tumor infiltration by V*δ*1^+^ T cells [11]. During HIV infection, V*δ*1^+^ T cell numbers are increased, thus suggesting the potential involvement of V*δ*1^+^ T cells in antiviral immunity [43].The dual antiviral and antitumor potential of this subset, analogous with the recognition of phopshoantigens in both cellular stress and in infection by *γ*9*δ*2 T cells [4, 8], suggests that the *in vitro* expansion of a parallel smaller population of V*δ*1 T cells might bring additional value to the cultured product.

The published studies on both *in vivo* and *in vitro* expansion of *γδ* T cells have, to the knowledge of the authors of this paper, almost exclusively focused on the *γ*9*δ*2 T cell subset, with one exception. In this study, *in vitro* expansion of *γδ* T cells was induced with anti-*γδ* TCR antibodies, and the resulting cell product contained both V*δ*2^+^ and V*δ*1^+^ T cells [46]. As the majority of the UCB *γδ* T cells at baseline are V*δ*1^+^ [16, 17], this approach may be interesting to explore also in the UCB setting in the future.

The spectratyping analysis indicated that the expanded cell populations cultured with zoledronate and varying concentrations of IL-2 (50–200 IU/ml) were mainly polyclonal (Figure 4). This indicates that the expanded *γδ* T cells, the majority of which expressed V*γ*9^+^ and V*δ*2^+^, had proliferated in a nonclonal, non-TCR specificity-dependent manner. This is in accordance with known data regarding the activation of V*δ*2^+^ T cells by phosphoantigens: a complex process independent of MHC molecules, including the binding of the CD277 receptor and resulting in nonclonal proliferation [47]. The wide range of TCR specificities in the expanded cells could increase the chance of efficiency of adoptive therapy schemes based on the current protocol.

The memory phenotype of the UCB *γδ* T cells was of interest to us, as it has been described that certain T cell subsets are more effective when used as adoptive therapy than others [48]. We initially utilized the most common T cell memory definition based on CCR7 and CD45RO. However, the baseline UCB and PB *γδ* T cell population had high percentages of terminally differentiated cells according to this definition (Figure 3(d)). Both human and animal model-based studies of skin *γδ* T cells have shown that this subset expresses low levels of CCR7 and that their recirculation between lymph nodes and skin is less dependent on CCR7 expression than the recirculation of *αβ* T cells [49, 50]. V*δ*2^+^ T cells have been described to express comparatively higher levels of CCR7 [51]. This might indicate that the standard definition for memory subsets is less suited to UCB *γδ* T cells. We then chose to further study a memory phenotype definition based on CD27 and CD45RO. This decision was based on the previous use of this definition in several published articles [39, 40]. The results with regard to the memory phenotype UCB *γδ* T cells before and after culture were then more in accordance with expectation: the majority of the UCB *γδ* T cells at baseline was naïve (CD45RO^−^ CD27^+^) and the largest subset after culture expressed a central memory phenotype (CD45RO^+^ CD27^+^, Figure 3(e)). The latter finding is reassuring, as this subset has been described to have the highest efficiency in vivo in a study of adoptive cell therapy with α*β* T cells in primates [48]. In contrast, PB *γδ* T cells expressed almost equal proportions of the CD27^−^ and CD45RO-based memory phenotypes, with slightly more naïve and central memory cells at baseline, while the dominant phenotype after culture was a CD27^−^ CD45RO^+^ effector memory phenotype [48].

Exploration of the cytokine gene expression at the end of culture indicated, interestingly, that there were differences in the expression patterns of UCB and PB *γδ* T cells. There was with significantly higher expression of IL-1*β*, IL-2, and IL-8 in UCB-derived cells (Figure 5(a). This might indicate a preferential development of a cytokine-producing phenotype, as has been previously reported [15]. IL-1*β* is a proinflammatory cytokine, which is released following activation of inflammosomes in response to viral infection [52], suggesting that UCB *γδ* T cells might have superior properties for mounting antiviral responses. IL-8 is known to cause chemotaxis and degranulation in neutrophil granulocytes [53], indicating a general proinflammatory capacity. The cytokine-producing phenotype did, however, not exclude cytotoxic ability. The cytotoxicity assay we performed indicated that the capacity for tumor cell killing was comparable in UCB and PB *γδ* T cells (Figures 5(b) and 5(c)). This is reassuring, as it indicates antitumor potential in UCB *γδ* T cell products cultured in this manner.

We saw downregulation of CD28 and upregulation of CD95, CD69 in cultured UCB and PB T cells, and upregulation of PD-1 in UCB T cells only, compared to baseline values (Figures 6(a) and 6(b)). This probably reflects activation [54, 55], indicating that the exposure to zoledronate and IL-2 led to successful antigen recognition-based stimulation. The fact that the percentage of CTLA-4^+^ and LAG-3^+^ T cells was significantly increased preferentially in UCB cells cultured with 50 IU IL-2/ml compared to baseline and to UCB T cells cultured with higher IL-2 concentrations (Figure 6(c)) may be due to expression kinetics or to the fact that the percentage of *γδ* T cells in cultures expanded with 50 IU IL-2/ml varied substantially between the UCB units used, and thus results might partly reflect marker expression in *αβ* T cells.

The cultured cell populations contained non-*γδ* T cells, consisting of *αβ* T cells and NK cells (Figure 6(d), left and middle panel). This might indicate that a final selection step could be introduced into the protocol, to ensure a pure *γδ* T cell product.

Interestingly, there was also a small fraction of CD56^+^ T cells present in after culture in the majority of expansions. As *γδ* T cells are known to express both CD56 and the NK cell-associated markers CD16 and NKG2D, these cells could be part of the expanded *γδ* T cell population. CD56 has been associated with cytotoxicity in previous studies of *in vitro γδ* T cell expansions [18].

In summary, the protocol for *in vitro* PB *γδ* T cell expansion here adapted to UCB induced substantial proliferation of UCB *γδ* T cells. Culture for 14 days in the preferred culture conditions, with 200 IU IL-2/ml combined with 5 *μ*M zoledronate in the culture medium, resulted in cell products with a high proportion of *γδ* T cells, mainly expressing *γ*9 chains and *δ*2 chains, in both UCB- and PBMC-derived cultures, and with a smaller population of remaining *δ*1 T cells possibly also expressing the *γ*9 chain, in UCB-derived cultures. The cultured UCB *γδ* T cells expressed a central memory phenotype. We could demonstrate that the cultured UCB *γδ* T cells were based on gene expression and efficient cytokine producers, especially with regard to IL-1*β*, IL-2, IL-8, and IL-15, and had a capacity for killing tumor cells comparable to cultured PB *γδ* T cells. Together, these results indicate that the development of *in vitro* expanded UCB *γδ* T cell therapies with this protocol is feasible and holds considerable potential, especially for ACT after UCBT.

## Figures and Tables

**Table 1:** Surface staining.

Target antigen	Fluorophore	Clone	Company, address
CD28	FITC	CD28.2	*BD Bioscience* Franklin Lakes, NJ, USA
CD56	FITC	NCAM16.2	
CD69	FITC	FN50	
CD94	FITC	HP-3D9	
CD95	FITC	DX2	
PD-1	FITC	MIH4	
PD-1	BV421	MIH4	
TCR*αβ*	FITC	T10B9.1A-31	
CD8	FITC	SK1	
CD8	APC Cy7	SK1	
CD8	V500	RPA-T8	
CD45RO	APC	UCHL1	
CD4	Alexa Fluor 700	RPA-T4	
CD3	PE	UCHT1	
CD3	V450	UCHT1	
CD3	BV510	UCHT1	
CD19	PE	HIB19	
CD27	PE	M-T271	
CD27	BV421	M-T271	
CCR7	PE-Cy7	3D12	
TCR*γδ*	FITC	IMMU510	*Beckman Coulter Inc.* Indianapolis, IN, USA
Cd152/CTLA-4	FITC	A3.4H2.H12	*LifeSpan Biosciences* Seattle, WA, USA
CD223/LAG-3	FITC	17B4	
CD366/TIM-3	APC	F38-2E2	
TCR V*δ*2	FITC	B6	*BioLegend* San Diego, CA, USA
TCR V*γ*9	FITC	B3	
TCR V*δ*1	FITC	TS8.2	*Thermo Fisher Scientific* Waltham, MA, USA
TCR*γδ*	PE	REA591	*Miltenyi* Bergisch Gladbach, Germany
*Apoptosis and viability*			
7AAD			*BD Biosciences*
Annexin V	FITC		
Annexin V	APC		*Immunotools GmbH* Friesoythe, Germany

7-AAD: 7-aminoactinomycin; APC: allophycocyanin; CD: cluster of differentiation; FITC: fluorescein isothiocyanate; PE: phycoerythrin.

## Supplementary Materials

Supplementary Materials The supplementary material includes 3 tables, Tables S1–3, and one figure, Supplementary Figure 1. The supplementary tables detail the setup of the spectratyping analysis with regard to analyzed *δ* chain subfamilies in Table S1, with regard to assessed *γ* subfamilies in Table S2, and for the primers used in Table S3. Supplementary Figure 1 displays the baseline proportions of *γδ* T cells, of *γδ* T cells positive for V*γ*9, V*δ*2, and V*δ*1, and of NK cells divided according to UCB donor sex. Cell subsets at baseline in UCB from male and female newborns. The proportions of key cell subsets at baseline are displayed separately for UCB units obtained from male and female donors. In A, the proportions of *γδ* T cells and NK cells are displayed, and in B, the percentages of *γδ* T cells positive for V*γ*9, V*δ*2, and V*δ*1 are shown.
