# Supplementary material for: Expansion of Gammadelta T Cells from Cord Blood: A Therapeutical Possibility
Source: Stem Cells Int. 2018 Mar 7;2018:8529104. doi: 10.1155/2018/8529104 (PMC5863314; doi:10.1155/2018/8529104)
Supplement: Supplementary Materials — The supplementary material includes 3 tables, Tables S1–3, and one figure, Supplementary Figure 1. The supplementary tables detail the setup of the spectratyping analysis with regard to analyzed δ chain subfamilies in Table S1, with regard to assessed γ subfamilies in Table S2, and for the primers used in Table S3. Supplementary Figure 1 displays the baseline proportions of γδ T cells, of γδ T cells positive for Vγ9, Vδ2, and Vδ1, and of NK cells divided according to UCB donor sex. Cell subsets at baseline in UCB from male and female newborns. The proportions of key cell subsets at baseline are displayed separately for UCB units obtained from male and female donors. In A, the proportions of γδ T cells and NK cells are displayed, and in B, the percentages of γδ T cells positive for Vγ9, Vδ2, and Vδ1 are shown. [file 8529104.f1.zip › Supplementary Material.docx]

Supplementary Tables

Table S1

| **TCR-δ tubes** | **V primer/s** | **J primer/s** | **Final primer conc.** |
| --- | --- | --- | --- |
| **1** | Vδ1 (FAM) | Jδ1 | 200 nM each |
|  | Vδ2 (NED) |  |  |
|  | Vδ3 (VIC) |  |  |
| **2** | Vδ1 (FAM) | Jδ2 | 200 nM each |
|  | Vδ2 (NED) |  |  |
|  | Vδ3 (VIC) |  |  |
| **3** | Vδ1 (FAM) | Jδ3 | 200 nM each |
|  | Vδ2 (NED) |  |  |
|  | Vδ3 (VIC) |  |  |
| **4** | Vδ1 (FAM) | Jδ4 | 400 nM each |
|  | Vδ2 (NED) |  |  |
|  | Vδ3 (VIC) |  |  |

Table S2

| **TCR-γ tubes** | **V primer/s** | **J primer/s** | **Final primer conc.** |
| --- | --- | --- | --- |
| **5** | Vγ10  VγfI | Jγ1.3/2.3 (NED)  Jγ1.1/2.1 (FAM) | 200 nM each |
| **6** | Vγ11 | Jγ1.3/2.3 (NED)  Jγ1.1/2.1 (FAM) | 400 nM each |
| **7** | Vγ9 | Jγ1.3/2.3 (NED)  Jγ1.1/2.1 (FAM) | 200 nM each |
| **8** | Vγ9 | Jγ1.2(FAM) | 400 nM each |

Table S3

| Primer | Sequence |
| --- | --- |
| Vδ1 | (FAM)- ATGCAAAAAGTGGTCGCTATT |
| Vδ2 | (NED)- ATACCGAGAAAAGGACATCTATG |
| Vδ3 | (VIC) GTACCGGATAAGGCCAGATTA |
| Jδ1 | GTTCCACAGTCACACGGGTTC |
| Jδ2 | GTTCCACGATGAGTTGTGTTC |
| Jδ3 | CTCACGGGGCTCCACGAAGAG |
| Jδ4 | TTGTACCTCCAGATAGGTTCC |
| VγfI* | GGAAGGCCCCACAGCRTCTT |
| Vγ10 | AGCATGGGTAAGACAAGCAA |
| Vγ9 | CGGCACTGTCAGAAAGGAATC |
| Vγ11 | CTTCCACTTCCACTTTGAAA |
| Jγ1.1/2.1 | (FAM)-TTACCAGGCGAAGTTACTATGAGC |
| Jγ1.3/2.3 | (NED)- GTGTTGTTCCACTGCCAAAGAG |
| Jγ1.2 | (FAM)-AAGAAAACTTACCTGTAATGATAAGC |

(*family)

Supplementary Figure legends

Supplementary Figure 1

Cell subsets at baseline in UCB from male and female newborns

The proportions of key cell subsets at baseline are displayed separately for UCB units obtained from male and female donors. In A, the proportions of γδ T cells and NK cells are displayed, and in B, the percentages of γδ T cells positive for Vγ9, Vδ2 and Vδ1 are shown. Abbreviations: NK: natural killer.
